# Lattice Radiation Therapy Plays a Synergistic Role in the Radical Treatment of Bulky Cervical Cancer: A Case Report and Literature Review

**DOI:** 10.3390/curroncol33040196

**Published:** 2026-03-31

**Authors:** Feifei Wang, Qianru Zhan, Zhitao Dai, Huijuan Zhang, Miao Peng, Zhijian Chen, Jing Jin, Xiugui Sheng

**Affiliations:** 1 Department of Radiation Oncology, National Cancer Center/National Clinical Research Center for Cancer/Cancer Hospital & Shenzhen Hospital, Chinese Academy of Medical Sciences and Peking Union Medical College, Shenzhen 518116, China; wangfeifei@cicams-sz.org.cn (F.W.);; 2 Department of Family Relations and Applied Nutrition, University of Guelph, Guelph, ON N1G 2W1, Canada; 3 Department of Pathology, National Cancer Center/National Clinical Research Center for Cancer/Cancer Hospital & Shenzhen Hospital, Chinese Academy of Medical Sciences and Peking Union Medical College, Shenzhen 518116, China

**Keywords:** locally advanced bulky cervical cancer, lattice radiation therapy, induction chemotherapy, concurrent chemoradiotherapy

## Abstract

Background: An increasing number of reports showed patients with bulky tumors after lattice radiation therapy (LRT) treatment achieved good local control. However, in these reports, LRT was previously used primarily for palliation. We reported a case that LRT plays a synergistic role in the radical treatment of locally advanced bulky cervical cancer (LABCC) combined with INTERLACE study protocol. Methods: The patient was a 54-year-old female with LABCC and treated with LRT combined with the INTERLACE study protocol. She received three fractions of 3 Gy each to the gross tumor volume (GTV) and three fractions of 9 Gy each to the lattice therapy volume (LTV), on an emergent basis, using volumetric modulated arc therapy (VMAT). Subsequently, according to the INTERLACE study protocol, chemotherapy and radiotherapy were carried out and the standard follow-up examinations were conducted. Adverse events (AEs) were assessed according to the Common Terminology Criteria for AEs (CTCAE) version 5.0. Results: The patient initially received LRT, which reduced the tumor burden and controlled bleeding. After this was combined with the INTERLACE study protocol, the complete clinical response (cCR) was achieved and they maintained this status for 13 months after the completion of concurrent chemoradiotherapy (CCRT), with only manageable grade IV hematological toxicity observed after the completion of CCRT. During this period, only manageable grade IV hematological toxicity (platelet count 16 × 10^9^/L, white blood cell count 0.33 × 10^9^/L) was observed. Conclusions: In this case, LRT combined with INTERLACE study protocol appears to be a safe and effective for the treatment of LABCC which improved the patient’s quality of life without uncontrolled treatment-related toxicity.

## Simple Summary

Bulky cervical cancer is extremely challenging to treat with traditional methods. Lattice radiation therapy was previously used primarily for palliation. We reported that lattice radiation therapy plays a synergistic role in the radical treatment of locally advanced bulky cervical cancer, with only manageable grade IV hematological toxicity observed. This patient initially received lattice radiation therapy, which significantly reduced the tumor burden and controlled bleeding, ultimately achieving a complete clinical response and they have maintained this status for 13 months to date. Lattice radiation therapy appears to be a safe and effective approach for the treatment of locally advanced bulky cervical cancer which improved the patient’s quality of life without uncontrolled treatment-related toxicity. We hope the case report will provide an alternative treatment option for locally advanced bulky cervical cancer, further improving the quality of life and overall survival of patients with bulky tumors.

## 1. Introduction

Cervical cancer is the second most common malignancy and the third highest cause of cancer mortality among women with malignant tumors worldwide [1]. The international federation of obstetrics and gynecology (FIGO) 2018 and the national comprehensive cancer network guidelines recommend platinum-based CCRT for stage IIB-IVA cervical cancer [2,3]. However, the guidelines do not specify the specific treatment plan for bulky tumors, especially for those with a diameter greater than 10 cm.

Stage T4 LABCC is extremely challenging to treat with traditional methods, especially for tumors larger than 10 cm in diameter that invade the surrounding tissues, such as the bladder, ureter, and rectum, etc. Hugert et al. [4] showed LABCC (>4 cm in diameter) was an increased incidence of treatment failure. Despite some studies showing that the overall survival (OS) of LABCC improved to a certain extent by adding induction chemotherapy, immunotherapy or new radiotherapy techniques to the standard treatment [5,6,7,8], bulky tumors usually present heterogeneity, hypoxia and an immunosuppressive microenvironment, which makes the tumors difficult to control [9].

LRT overcomes the dose limitations of stereotactic body radiation therapy (SBRT) which possibly achieves a more durable tumor response and is particularly suitable for bulky tumors (≥5.0 cm) [10]. The principle is based on distributing high-dose areas, called vertices, within the central area of the GTV, and lower dose areas, called valleys, also within the GTV. This high-dose radiation is delivered to bulk tumors, while sparing the surrounding areas and normal tissues [11,12]. The valley-to-peak dose ratio of LRT is defined by the ratio of the valley doses, called lower doses—cold spots—to the peak doses, called higher doses—hot spots. The valley-to-peak ratio thereby quantifies the degree of spatial fractionation [13].

When the tumor was delivered ablation dose radiotherapy, the lysis and death of the tumor cells released tumor-associated antigens (TAA), which could improve the infiltration of immune cells in the tumor tissue and activate an anti-tumor immune response [14,15]. Animal experiments showed LRT promoted tumor growth inhibition by generating anti-tumor inflammatory factors, improving immune cell infiltration, and increasing the expression of tumor necrosis factor-related apoptosis-inducing ligand (TRAIL) [16]. Furthermore, some clinical studies also showed LRT achieved good results in treating advanced bulky tumors and held great clinical potential for treating advanced bulky tumors combined with immunotherapy [14,17,18].

## 2. Case Presentation

A 54-year-old female patient was menopausal at the age of 50, with a history of thalassemia, penicillin allergy and chronic hepatitis B virus infection. Two years before the treatment, she presented with irregular vaginal bleeding without obvious causes and was not diagnosed and treated. The irregular vaginal bleeding was aggravated 2 months later, and the pathological result of cervical biopsy showed cervical squamous cell carcinoma with moderate differentiation. Due to fear of chemotherapy and radiotherapy, the patient chose traditional Chinese medicine and herbal therapy, and considered that the vaginal bleeding had improved compared to before.

Six months before the treatment, the vaginal bleeding had worsened and the positron emission tomography-computed tomography (PET-CT) showed the size of the cervical mass was approximately 6.5 cm × 5.9 cm × 9.0 cm, invading the uterine and the upper 2/3 of the vagina. Fluorodeoxyglucose F-18 (FDG) uptake was diffusely and unevenly increased, with a SUV max of 11.1. It also invaded the bladder wall and the left ureter. The left renal pelvis and ureter were dilated and filled with fluid. No obvious enlarged lymph nodes or metastatic lesions were observed throughout the body.

**Figure 1:**
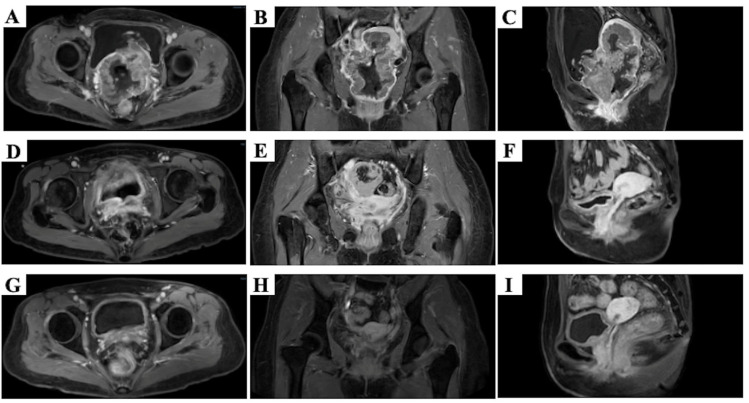
The images of pelvic MRI in an axial plane (**A**,**D**,**G**), in a coronal plane (**B**,**E**,**H**) and in a sagittal plane (**C**,**F**,**I**). Before treatment (**A**–**C**), after LRT and 2 cycles of induction chemotherapy (**D**–**F**) and 3 months after the completion of CCRT (**G**–**I**).

During the month of treatment, the patient experienced continuous vaginal bleeding with foul odor and poor physical condition. The hematologic tests showed anemia, increased levels of creatinine (Cr) and squamous cell carcinoma antigen (SCC), electrolyte imbalance, increased inflammatory markers, urinary tract infection, positive hepatitis B surface antibody, and a quantitative determination of hepatitis B virus DNA at 1.15 × 10^6^ IU/mL, while the level of white blood cells (WBC) was normal. Adverse events (AEs) were assessed according to CTCAE version 5.0. The pelvic magnetic resonance imaging (MRI) showed a full-circumferential mass in the cervix, approximately 9.1 cm × 7.5 cm × 10.3 cm, which was invading the uterus, the lower 1/3 of vagina, the mucosa layer of the bladder, the upper 2/3 of the urethra and the bilateral ureters, which were dilated and filled with fluid (Figure 1A–C). Furthermore, no obvious evidence of metastasis was found in the neck, chest, abdomen and pelvis by computed tomography (CT). Therefore, the stage was IVA (FIGO 2018) [2].

The patient’s initial condition upon admission was poor. Therefore, the symptomatic treatment was executed, including the correction of anemia and the electrolyte imbalance, anti-infection and anti-hepatitis B virus. The Cr level was continuously rising due to the tumor invading the bilateral ureters, and a percutaneous bilateral renal fistula for drainage was performed on day 5.

**Figure 2:**
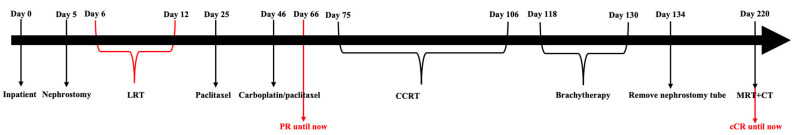
The management plan schematic diagram.

In order to achieve hemostasis and reduce the tumor burden, she was treated with LRT to create a nonhomogeneous dose distribution analogous to intracavitary/interstitial brachytherapy (IC/ISBT). LRT was performed from day 6 to day 12 (Figure 2). Considering the elevated hepatitis B virus DNA copy number and renal insufficiency and PD-L1 (CPS < 1), we administered one cycle of 130-milligram-per-square-meter paclitaxel induction chemotherapy on day 25 (Figure 2). On day 46, the levels of Cr, and the anemia and hepatitis B virus DNA copies improved, but there was a slight increase in liver transaminase and a weight loss of 6.5 kg over the course of one month. We provided symptomatic liver-protective treatment, nutritional support, and administered the second cycle of the TC regimen induction chemotherapy (paclitaxel 130 mg/m^2^ + carboplatin, area under the curve (AUC) = 4) on day 66 (Figure 2).

After LRT and two cycles of induction chemotherapy, MRI showed the cervical tumor had significantly shrunk and was approximately 3.2 cm × 1.3 cm × 2.0 cm, which was not invading the lower 1/3 of the vagina (Figure 1D–F). The efficacy evaluation indicated a partial response (PR). From day 75 to day 130, CCRT (carboplatin, AUC = 2 weekly) and brachytherapy were carried out (Figure 2). At this point, the cumulative dose of EQD2Gy for the cervical tumor was 84.82 Gy. The bilateral nephrostomy drainage tubes were removed after the completion of CCRT. However, due to the grade IV myelosuppression and urinary tract infections, we reduced the concurrent carboplatin by one dose, and the radiotherapy was also suspended for 3 days. At the time when 23 fractions of external beam radiotherapy were carried out, the mid-term follow-up pelvic MRI showed the cervical tumor had shrunk further, approximately to 2.4 cm × 1.1 cm × 2.0 cm, and invaded the upper 2/3 of the vagina. In addition, we also explored the ratios of CD3+ T cells, CD4+ T cells and CD8+ T cells in peripheral blood. Before the treatment, the flow cytometry test results showed the ratios of CD3+ T cells, CD4+ T cells, CD8+ T cells, and CD4+ T cells/CD8+ T cells were 80%, 37%, 39%, and 0.95%, which increased to 87.78%, 43.78%, 40.32% and 1.09% during the CCRT, respectively.

One month after the completion of CCRT, MRI showed the cervical tumor was not clearly visible and approximately 1.1 cm of the thickest part was not invading the vagina and uterus. In addition, the hematologic tests showed Hb: 107 g/L, Cr: 87 μmol/L, and both WBC and SCC were at normal levels.

Three months after the completion of CCRT, no obvious tumor was observed in the mages of the MRI (Figure 1G–I). Furthermore, no obvious evidence of recurrence and distant metastasis was observed in the CT of the neck, thoracic and abdominal area during the treatment and follow-up. The efficacy evaluation indicated a cCR. Moreover, the hematologic tests showed Hb had returned to normal level, and WBC and SCC remained within the normal range. Only the level of Cr slightly increased to 92 μmol/L. The re-examination results indicated that the patient remained in cCR and has maintained this status for 13 months up to now.

### 2.1. LRT

**Figure 3:**
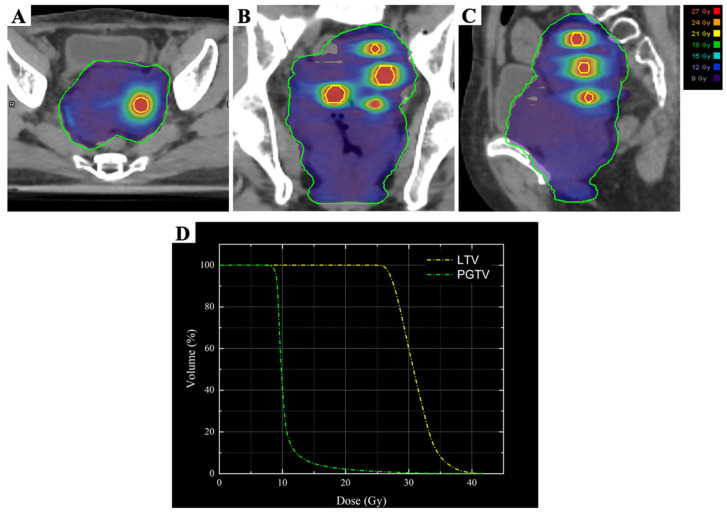
Three-dimensional dose distribution and dose–volume histograms (DVHs) for the present case. The dose distributions are shown in the axial (**A**), coronal (**B**), and sagittal (**C**) planes, and the DVHs are shown in (**D**). The lattice spheres are delineated by the yellow contours, and PGTV is outlined in green. The D95% values for LTV and PGTV were 27 Gy and 9 Gy, respectively.

According to Wu et al.’s description [19], the target volume and organs at risk (OARs) were contoured in combination with the positioning CT: GTV was the MRI-visible gross tumor, LTV was the four high-dose vertices of the tumor, avoiding the vaginal wall to prevent the formation of a vaginal fistula (Figure 3A), and the planning gross tumor volume (PGTV) was obtained by expanding in three dimensions with a 5 mm margin around GTV. The radiotherapy plan: VMAT technology, 6MV-X rays, 95% PGTV 9 Gy/3 Gy/3f, LTV 27 Gy/9 Gy/3f, with every other day treatment. Meanwhile, the dose limitation of OARs were implemented based on Timmerman 2021 [20]. The cumulative doses of EQD2 Gy Max for the rectum, bladder, sigmoid, and small intestine were 11.64 Gy, 11.90 Gy, 11.66 Gy and 11.55 Gy, respectively (Table 1).

### 2.2. External Beam Radiation Therapy (EBRT)

The target volume contour: The low-risk clinical target volume (CTV_LR) included the uterus, cervix, entire vagina, common iliac arteries, bilateral internal iliac arteries, bilateral external iliac arteries, bilateral obturator, part of the pre-sacral region, bilateral inguinal regions, and the 3 cm para-aortic lymph node drainage areas. The low-risk planning tumor volume (PTV_LR) was obtained by expanding in three dimensions with a 5 mm margin around CTV_LR. Normal organs were also contoured. The adaptive planning was used during EBRT and it was reviewed and modified. The radiotherapy plan: VMAT technique, 6MV-X rays, 95% PTV_LR 45 Gy/1.8 Gy/25 fractions. The OARs are implemented in accordance with the recommendation of QUANTEC [21]. The cumulative doses of EQD2Gy Max for the rectum, bladder, sigmoid, and small intestine are 44.62 Gy, 46.85 Gy, 45.78 Gy and 46.33 Gy, respectively (Table 1).

### 2.3. IC/ISBT

During the IC/ISBT process, the pain relief was administered before the tandem applicator was placed, and the position of two interstitial needles were confirmed under CT guidance. The target volume contour: the high-risk clinical target volume (CTV_HR) represents cervical and imaging residual lesions. The intermediate-risk clinical target volume CTV_IR is the expansion of CTV_HR by 1 cm in the up–down and lateral directions around the uterus, and by 0.5 cm in the front–back direction, and it includes the range of tumor invasion before EBRT. The IC/ISBT was performed in a total of four fractions, and the D90 doses for each fraction were 5.74, 5.7, 5.56, and 6.34, respectively, totaling 23.35 Gy. The target volume contour and the dose limits for OARs were carried out in accordance with the International Commission on Radiation Units and measurements (ICRU) report 89 [22]. The cumulative doses of EQD2Gy Max for the rectum, bladder, sigmoid, and small intestine are 17.99 Gy, 25.33 Gy, 8.76 Gy and 17.07 Gy, respectively (Table 1).

## 3. Discussion

Spatial fractionated radiotherapy (SFRT) is one of the cutting-edge technologies in the field of tumor radiotherapy and emphasizes highly non-uniform irradiation of the tumor area in three-dimensional space, which can break through the dose limitations of traditional radiotherapy and achieve the dual benefits of tumor control and normal tissue protection. It is particularly suitable for the treatment of large-volume malignant tumors [23,24]. LRT is one type of SFRT and has the potential to improve outcomes by delivering high-dose vertices within the tumor to trigger the bystander effect [19]. An increasing number of reports showed patients with bulky tumors after LRT treatment achieved good local control [14,25,26,27,28]. However, in these reports, LRT was previously used primarily for palliation. In the present case presentation, we reported that LRT plays a synergistic role in the radical treatment of bulky cervical squamous cell carcinoma, with only manageable grade IV hematological toxicity observed. According to the literature we have reviewed, this case had the larger tumor volume among those treated with LRT at present.

The KEYNOTE-A18 study introduced immunotherapy into the CCRT standard treatment and increased the 3-year OS rate of LACC from 74.8% to 82.6% [6,7]. The INTERLACE study achieved almost the same 3-year OS to the KEYNOTE-A18 study [5]. The EMBRACE-I study introduced MR guidance during brachytherapy following the standard CCRT [29], and may have encountered similar side effects to the KEYNOTE-A18 and INTERLACE studies in the treatment of LABCC. Therefore, it is necessary to integrate existing studies to enhance the therapeutic effect and reduce the treatment toxicity. For LABCC, Saito et al. reported a case achieved good therapeutic results with chemoradiotherapy, regional hyperthermia and interstitial brachytherapy [30]. However, this case had a poor physical condition and was also experiencing continuous vaginal bleeding, which led to the limitations of this treatment model.

LRT has both dosimetric and biological advantages for treating bulky tumors, which are determined by the vertex volume diameter and center-to-center spacing. Gholami et al. [31] found that the hot spots diameters of 1 to 1.25 cm, separated by 1.7 to 1.8 cm, are key to optimize the therapeutic ratio and normal tissue sparing. Yuan et al. [32] reported that biologically guided LRT (BG-LRT) significantly improved peak–valley dose ratio (PVDR) and ablation dose ratio (ADR) in GTV, focusing on dose escalation in biologically relevant tumor regions. This technique maintains low OARs doses and represents a promising step toward personalized LRT planning. Additionally, Ferini et al. [33] demonstrated favorable clinical outcomes in multicenter studies of PET-CT-guided LRT for bulky tumors. In terms of the advantages of image-guided LRT, DWI offered significant advantages as a biological guidance tool and was more economical [34]. But, DWI had lower spatial resolution and signal-to-noise ratio [17]. Therefore, it is necessary to focus on the value of multiple parameters image-guided LRT in future research.

In today’s era of multidisciplinary comprehensive treatment, single LRT is far from sufficient. Some clinical and basic studies have shown that LRT combined with immunotherapy achieves good local control effects for advanced large-volume tumors. However, the existing research results are limited to small sample reports and lack scientific, systematic, and large-sample clinical research data support [14,17,28,35]. According to the information we have obtained, many medical centers in China, including our center, are currently conducting clinical research related to LRT. We look forward to more clinical research data reports in the future, providing more clinical and basic research support for LRT.

In the present case, where the patient initially experienced continuous vaginal bleeding, we used LRT with two purposes: hemostasis and reducing the tumor volume to lower the dose of OARS in the subsequent EBRT. Just as we expected, the vaginal bleeding was significantly controlled afterwards and the tumor volume significantly decreased and reached PR. Ultimately, the tumor achieved cCR 3 months after the completion of CCRT. It should be noted that while LRT and conventional radiotherapy were administered in a sequential manner, LRT may have triggered a cumulative bystander effect rather than a strong bystander effect, and chemotherapy also plays a certain role in achieving a synergistic anti-tumor effect. Whether LRT triggered the anti-tumor immune response still requires further research.

Although MRI showed that the tumor had invaded the bladder before the treatment, which indicated a risk of vaginal fistula, we did not observe the adverse reaction through the treatment and follow-up, suggesting that LRT is a safe radiotherapy technique. The case demonstrates that LRT could play a synergistic role in the radical treatment of T4 stage bulky cervical cancer. However, since this is the only single case treated by our center using this technology, and it is the result observed only 13 months after the completion of CCRT, we still cannot ignore the management of the patients. Further research is still needed to verify the safety and effectiveness of this technique. Finally, safe and effective treatment not only requests higher demands on radiation therapy equipment, but also relies on the collaboration of the entire team, including doctors, physicists, therapists and nurses.

## 4. Conclusions

In preliminary experience with the present case, LRT combined with INTERLACE study protocol treatment for the case of bulky cervical tumors significantly reduced the tumor volume and effectively stopped the bleeding, improving the patient’s quality of life without uncontrolled treatment-related toxicity. Further prospective clinical trials need to be developed in order to test this hypothesis.

## Figures and Tables

**Table 1:** During the processes of LRT, EBRT and IC/ISBT, the rectum, bladder, sigmoid and small intestine received the cumulative dose of the EQD2Gy Max. The α/β ratio for normal tissues is 3.

	LRT	EBRT	IC/ISBT			
total dose	27	45	5.74	5.70	5.56	6.34
fraction	3	25	1	1	1	1
rectum	9.46	46.07	3.44	3.64	3.51	3.30
bladder	9.60	47.72	4.28	4.01	4.52	4.47
sigmoid	9.47	46.93	2.04	1.91	2.27	2.30
small intestine	9.41	47.34	3.64	3.27	2.89	3.59
EQD2						
rectum	11.64	44.62	4.43	4.83	4.57	4.16
bladder	11.90	46.85	6.23	5.62	6.80	6.68
sigmoid	11.66	45.78	2.06	1.88	2.39	2.44
small intestine	11.55	46.33	4.83	4.10	3.40	4.73

## Data Availability

The original contributions presented in this study are included in the article. Further inquiries can be directed to the corresponding authors.

## References

[1] Arbyn M., Weiderpass E., Bruni L., De Sanjosé S., Saraiya M., Ferlay J., Bray F. (2020). Estimates of incidence and mortality of cervical cancer in 2018: A worldwide analysis. Lancet Glob. Health.

[2] Wright J.D., Matsuo K., Huang Y., Tergas A.I., Hou J.Y., Khoury-Collado F., Clair C.M.S., Ananth C.V., Neugut A.I., Hershman D.L. (2019). Prognostic Performance of the 2018 International Federation of Gynecology and Obstetrics Cervical Cancer Staging Guidelines. Obstet. Gynecol..

[3] Abu-Rustum N.R., Yashar C.M., Arend R., Barber E., Bradley K., Brooks R., Campos S.M., Chino J., Chon H.S., Crispens M.A. (2023). NCCN Guidelines® Insights: Cervical Cancer, Version 1.2024: Featured Updates to the NCCN Guidelines. J. Natl. Compr. Cancer Netw..

[4] Huguet F., Cojocariu O.-M., Levy P., Lefranc J.-P., Darai E., Jannet D., Ansquer Y., Lhuillier P.-E., Benifla J.-L., Seince N. (2008). Preoperative concurrent radiation therapy and chemotherapy for bulky stage IB2, IIA, and IIB carcinoma of the uterine cervix with proximal parametrial invasion. Int. J. Radiat. Oncol..

[5] McCormack M., Eminowicz G., Gallardo D., Diez P., Farrelly L., Kent C., Hudson E., Panades M., Mathew T., Anand A. (2024). Induction chemotherapy followed by standard chemoradiotherapy versus standard chemoradiotherapy alone in patients with locally advanced cervical cancer (GCIG INTERLACE): An international, multicentre, randomised phase 3 trial. Lancet.

[6] Lorusso D., Xiang Y., Hasegawa K., Scambia G., Leiva M., Ramos-Elias P., Acevedo A., Sukhin V., Cloven N., Pereira De Santana Gomes A.J. (2024). Pembrolizumab or placebo with chemoradiotherapy followed by pembrolizumab or placebo for newly diagnosed, high-risk, locally advanced cervical cancer (ENGOT-cx11/GOG-3047/KEYNOTE-A18): A randomised, double-blind, phase 3 clinical trial. Lancet.

[7] Lorusso D., Xiang Y., Hasegawa K., Scambia G., Leiva M., Ramos-Elias P., Acevedo A., Cvek J., Randall L., Pereira de Santana Gomes A.J. (2024). Pembrolizumab or placebo with chemoradiotherapy followed by pembrolizumab or placebo for newly diagnosed, high-risk, locally advanced cervical cancer (ENGOT-cx11/GOG-3047/KEYNOTE-A18): Overall survival results from a randomised, double-blind, placebo-controlled, phase 3 trial. Lancet.

[8] Knoth J., Nout R.A., Pötter R., Mahantshetty U., Jürgenliemk-Schulz I., Haie-Meder C., Fortin I., Fokdal L.U., Sturdza A., Hoskin P. (2025). Distant Metastasis After Chemoradiation and Image Guided Adaptive Brachytherapy in Locally Advanced Cervical Cancer. Int. J. Radiat. Oncol..

[9] Ferini G., Valenti V., Tripoli A., Illari S.I., Molino L., Parisi S., Cacciola A., Lillo S., Giuffrida D., Pergolizzi S. (2021). Lattice or Oxygen-Guided Radiotherapy: What If They Converge? Possible Future Directions in the Era of Immunotherapy. Cancers.

[10] Iori F., Cappelli A., D’Angelo E., Cozzi S., Ghersi S.F., De Felice F., Ciammella P., Bruni A., Iotti C. (2023). Lattice radiation therapy in clinical practice: A systematic review. Clin. Transl. Radiat. Oncol..

[11] Neuner G., Mohiuddin M.M., Vander Walde N., Goloubeva O., Ha J., Yu C.X., Regine W.F. (2012). High-Dose Spatially Fractionated GRID Radiation Therapy (SFGRT): A Comparison of Treatment Outcomes With Cerrobend vs. MLC SFGRT. Int. J. Radiat. Oncol..

[12] Blanco Suarez J.M., Amendola B.E., Perez N., Amendola M., Wu X. (2015). The use of lattice radiation therapy (LRT) in the treatment of bulky tumors: A case report of a large metastatic mixed mullerian ovarian tumor. Cureus.

[13] Billena C., Khan A.J. (2019). A Current Review of Spatial Fractionation: Back to the Future?. Int. J. Radiat. Oncol..

[14] Jiang L., Li X., Zhang J., Li W., Dong F., Chen C., Lin Q., Zhang C., Zheng F., Yan W. (2020). Combined high-dose LATTICE radiation therapy and immune checkpoint blockade for advanced bulky tumors: The concept and a case report. Front. Oncol..

[15] Li X.-N., Wang F., Chen K., Wu Z., Zhang R., Xiao C., Zhao F., Wang D., Zhao H., Ran Y. (2025). XCL1-secreting CEA CAR-T cells enhance endogenous CD8+ T cell responses to tumor neoantigens to confer a long-term antitumor immunity. J. Immunother. Cancer.

[16] Johnsrud A.J., Jenkins S.V., Jamshidi-Parsian A., Quick C.M., Galhardo E.P., Dings R.P.M., Vang K.B., Narayanasamy G., Makhoul I., Griffin R.J. (2020). Evidence for early stage anti-tumor immunity elicited by spatially fractionated radiotherapy-immunotherapy combinations. Radiat. Res..

[17] Duriseti S., Kavanaugh J.A., Szymanski J., Huang Y., Basarabescu F., Chaudhuri A., Henke L., Samson P., Lin A., Robinson C. (2022). LITE SABR M1: A phase I trial of lattice stereotactic body radiotherapy for large tumors. Radiother. Oncol. J. Eur. Soc. Ther. Radiol. Oncol..

[18] Luke J.J., Onderdonk B.E., Bhave S.R., Karrison T., Lemons J.M., Chang P., Zha Y., Carll T., Krausz T., Huang L. (2020). Improved survival associated with local tumor response following multisite radiotherapy and pembrolizumab: Secondary analysis of a phase I trial. Clin. Cancer Res. Off. J. Am. Assoc. Cancer Res..

[19] Wu X., Perez N.C., Zheng Y., Li X., Jiang L., Amendola B.E., Xu B., Mayr N.A., Lu J.J., Hatoum G.F. (2020). The technical and clinical implementation of LATTICE radiation therapy (LRT). Radiat. Res..

[20] Timmerman R. (2022). A story of hypofractionation and the table on the wall. Int. J. Radiat. Oncol..

[21] Bentzen S.M., Constine L.S., Deasy J.O., Eisbruch A., Jackson A., Marks L.B., Ten Haken R.K., Yorke E.D. (2010). Quantitative analyses of normal tissue effects in the clinic (QUANTEC): An introduction to the scientific issues. Int. J. Radiat. Oncol. Biol. Phys..

[22] (2013). International Commission on Radiation Units and Measurements in collaboration with Groupe Europeen de Curietherapie-European Society for Radiotherapy and Oncology. Prescribing, recording, and reporting brachytherapy for cancer of the cervix. J. ICRU.

[23] Misa J., Volk A., Bernard M.E., Clair W.S., Pokhrel D. (2024). Dosimetric impact of intrafraction patient motion on MLC-based 3D-conformal spatially fractionated radiation therapy treatment of large and bulky tumors. J. Appl. Clin. Med. Phys..

[24] Li H., Mayr N.A., Griffin R.J., Zhang H., Pokhrel D., Grams M., Penagaricano J., Chang S., Spraker M.B., Kavanaugh J. (2024). Overview and recommendations for prospective multi-institutional spatially fractionated radiation therapy clinical trials. Int. J. Radiat. Oncol..

[25] Iori F., Botti A., Ciammella P., Cozzi S., Orlandi M., Iori M., Iotti C. (2022). How a very large sarcomatoid lung cancer was efficiently managed with lattice radiation therapy: A case report. Ann. Palliat. Med..

[26] Liu X., Xu K., Yao Q., Fang J., Zhou W., Lang J. (2025). Metastatic colon adenocarcinoma to the gingiva treated with spatial fractionation radiotherapy: A case report. Front. Oncol..

[27] Iati’ G., Parisi S., Ferrantelli G., Pergolizzi S. (2025). Metabolism-guided LATTICE radiotherapy in an elderly patient with locally advanced head and neck cancer treated with curative aim: A case report. Reports.

[28] Hobson J., Grams M.P., Gicobi J.K., Wigle D., Kottschade L.A., Durham L.A., Corbin K., Dong H., Markovic S.N., Park S.S. (2025). Case report: Complete pathologic response in advanced melanoma with SFRT and dual checkpoint inhibition. Front. Oncol..

[29] Vittrup A.S., Kirchheiner K., Pötter R., Fokdal L.U., Jensen N.B.K., Spampinato S., Haie-Meder C., Schmid M.P., Sturdza A.E., Mahantshetty U. (2023). Overall severe morbidity after chemo-radiation therapy and magnetic resonance imaging-guided adaptive brachytherapy in locally advanced cervical cancer: Results from the EMBRACE-I study. Int. J. Radiat. Oncol..

[30] Saito T., Murakami M., Sumiya T., Kobayashi D., Shirataki H., Fujioka D., Baba K., Itagaki H., Tenjimbayashi Y., Satoh T. (2023). Multimodal treatment with chemoradiotherapy, regional hyperthermia and interstitial brachytherapy for a huge locally advanced cervical cancer: A case report. Tech. Innov. Patient Support Radiat. Oncol..

[31] Nedaie H., Gholami S., Longo F., Ay M., Dini S., Meigooni A. (2017). Grid block design based on monte carlo simulated dosimetry, the linear quadratic and hug–kellerer radiobiological models. J. Med. Phys..

[32] Yuan K., Zhou X., Guo H., Peng Y., Xu P., Li L., Li J., Li H., Lu S., Feng M. (2025). Feasibility study of functional magnetic resonance imaging-based biologically-guided lattice radiotherapy. Int. J. Radiat. Oncol..

[33] Ferini G., Parisi S., Lillo S., Viola A., Minutoli F., Critelli P., Valenti V., Illari S.I., Brogna A., Umana G.E. (2022). Impressive results after “metabolism-guided” lattice irradiation in patients submitted to palliative radiation therapy: Preliminary results of LATTICE_01 multicenter study. Cancers.

[34] Liu F., Wang H., Jiang C., He L., Xiao S., Ye X., Fan C., Wu X., Liu W., Li Y. (2023). Dose painting radiotherapy guided by diffusion-weighted magnetic resonance vs. 18F-FDG-PET/CT in locoregionally advanced nasopharyngeal carcinoma: A randomized, controlled clinical trial. Int. J. Radiat. Oncol..

[35] Leibfarth S., Winter R.M., Lyng H., Zips D., Thorwarth D. (2018). Potentials and challenges of diffusion-weighted magnetic resonance imaging in radiotherapy. Clin. Transl. Radiat. Oncol..

